# Exploitation of Heterosis in Pearl Millet: A Review

**DOI:** 10.3390/plants9070807

**Published:** 2020-06-27

**Authors:** Rakesh K. Srivastava, Srikanth Bollam, Vijayalakshmi Pujarula, Madhu Pusuluri, Ram B. Singh, Gopi Potupureddi, Rajeev Gupta

**Affiliations:** International Crops Research Institute for the Semi-Arid Tropics (ICRISAT), Hyderabad TS 502324, India; bm.srikanth@cgiar.org (S.B.); v.lakshmi@cgiar.org (V.P.); m.pusuluri@cgiar.org (M.P.); s.rambaran@cgiar.org (R.B.S.); p.gopi@cgiar.org (G.P.)

**Keywords:** heterosis, hybrid vigor, pearl millet, genome sequence, heterotic gene pools, genomic selection

## Abstract

The phenomenon of heterosis has fascinated plant breeders ever since it was first described by Charles Darwin in 1876 in the vegetable kingdom and later elaborated by George H Shull and Edward M East in maize during 1908. Heterosis is the phenotypic and functional superiority manifested in the F_1_ crosses over the parents. Various classical complementation mechanisms gave way to the study of the underlying potential cellular and molecular mechanisms responsible for heterosis. In cereals, such as maize, heterosis has been exploited very well, with the development of many single-cross hybrids that revolutionized the yield and productivity enhancements. Pearl millet (*Pennisetum glaucum* (L.) R. Br.) is one of the important cereal crops with nutritious grains and lower water and energy footprints in addition to the capability of growing in some of the harshest and most marginal environments of the world. In this highly cross-pollinating crop, heterosis was exploited by the development of a commercially viable cytoplasmic male-sterility (CMS) system involving a three-lines breeding system (A-, B- and R-lines). The first set of male-sterile lines, i.e., Tift 23A and Tift18A, were developed in the early 1960s in Tifton, Georgia, USA. These provided a breakthrough in the development of hybrids worldwide, e.g., Tift 23A was extensively used by Punjab Agricultural University (PAU), Ludhiana, India, for the development of the first single-cross pearl millet hybrid, named Hybrid Bajra 1 (HB 1), in 1965. Over the past five decades, the pearl millet community has shown tremendous improvement in terms of cytoplasmic and nuclear diversification of the hybrid parental lines, which led to a progressive increase in the yield and adaptability of the hybrids that were developed, resulting in significant genetic gains. Lately, the whole genome sequencing of Tift 23D_2_B_1_ and re-sequencing of circa 1000 genomes by a consortium led by the International Crops Research Institute for the Semi-Arid Tropics (ICRISAT) has been a significant milestone in the development of cutting-edge genetic and genomic resources in pearl millet. Recently, the application of genomics and molecular technologies has provided better insights into genetic architecture and patterns of heterotic gene pools. Development of whole-genome prediction models incorporating heterotic gene pool models, mapped traits and markers have the potential to take heterosis breeding to a new level in pearl millet. This review discusses advances and prospects in various fronts of heterosis for pearl millet.

## 1. Introduction

Heterosis (syn hybrid vigor) is a natural phenomenon whereby hybrid (first filial generation, i.e., F_1_) offsprings of genetically diverse individuals exhibit improved physical and functional features relative to their parents [1,2]. Heterosis has been studied in most eukaryotic organisms, including plants, animals, and fungi. When two homozygous inbred lines (true breeding line derived from recurrent inbreeding) with distinct genetic constitutions are hybridized together, the resultant hybrids have greater height, weight, fertility, robustness, and constitutional vigor than either of the parents and their self-pollinated counterparts [3]. Naturally cross-pollinating species like pearl millet, rye, maize, and other grasses typically exhibit a higher degree of heterosis than the self-pollinating crop plants such as rice, barley, wheat, and oats. Nevertheless, many hybrid cultivars have also been developed in self-pollinating plant species [4]. Heterosis can manifest by virtue of improvement of several traits during plant growth and development. In pearl millet, considerable growth differences between hybrids and their parents can be monitored during different stages of growth and development. Post-germination, two root architectural traits such as primary root length and lateral root density show differences at early as well as later stages. During late development stages, the F_1_ hybrids display relatively more luxurious growth, greater biomass accumulation, and higher seed setting than their parent genotypes. It is noteworthy that the degree of heterosis can vary considerably among various traits. For example, grain iron (Fe) and zinc (Zn) contents in pearl millet F_1_ hybrids do not improve over their parental lines and require high levels of micronutrients in both parents. The maximum level of heterosis is monitored in the F_1_ derived from cross-pollination between diverse genotypes. This demonstrates the involvement of several alleles or genetic loci with diverse interactions at cellular and molecular levels. While the superiority of the progenies over their parents is progressively reduced in the successive generations developed from self-pollination. Heterosis has been exploited in many cereals, oilseeds, vegetables, fruits, pulses, etc., through the development of F_1_ hybrids globally [4]. 

### The Evolution of Understanding of Heterosis

During the early era, genetic research on “heterosis” and inheritance among inter-accession crosses from different progenitors was explored by Darwin [3] and Mendel [5]. Heterosis has some salient characteristics, such as its high variability, with the degree of heterosis differing according to the genetic background of the parents, their reproductive mode (vegetative or sexual), the traits under consideration [6], developmental stage of the plant (vegetative and grain filling) [7], the surrounding environments (moisture content, light intensity, CO_2_ level, and heat) [8], etc.

Heterosis was first observed in tobacco hybrids by Koelreuter (1733–1806) [9], and further, it was scientifically characterized by many researchers in different crop species [2,3,10,11,12]. Because of known biological and economic precedence, the underlying genetic mechanisms regulating heterosis have fascinated the research community for over a century. Genetic mechanisms for heterosis may vary greatly across different species and are indeed dependent upon their mode of pollination, whether naturally self-pollinating or by out-crossing. Heterosis is more prevalent in outcrossing species than in inbreeding species; indeed, these inbreeding populations do not have complete heterosis of fitness [13]. This indicates that in a genetic out-cross, the interaction between different alleles in the F_1_ generation makes them outperformed when compared to the effect of homozygous genotypes in the inbred parental lines [14]. Even though the superior performance of F_1_ heterozygous hybrids over inbred parental homozygous lines has been well documented, continuous self-pollination of these hybrids for several generations will lead to inbreeding depression [15]. Several clues from the genetic studies describe that the vigor of hybrid is due to the genomic shock or genomic turbulence event, generated during the union of two discrete genomes which may further lead to whole genome-wide relaxation, to deliver novel/differential gene expression patterns in hybrids [16,17]. In the majority of cases, spatio-temporal gene expression patterns may alter the degree of heterosis across the plant from the vegetative to the reproductive phase [18]. Despite these superficial observations on crop heterosis, the underlying genetic, molecular and biochemical mechanisms are still to be unraveled to some extent. Moreover, crop species like pearl millet with an out-crossing rate above 85% will certainly be useful for developing open-pollinated varieties (OPVs) and single-cross hybrids for the exploitation of heterosis [19]. 

Building on our classical understanding of the phenomenon of manifestation of heterosis, several studies have been performed to dissect the underlying causal mechanisms at cellular and molecular levels. Some of these are discussed in the following section.

## 2. Molecular Bases of Heterosis

**Figure 1:**
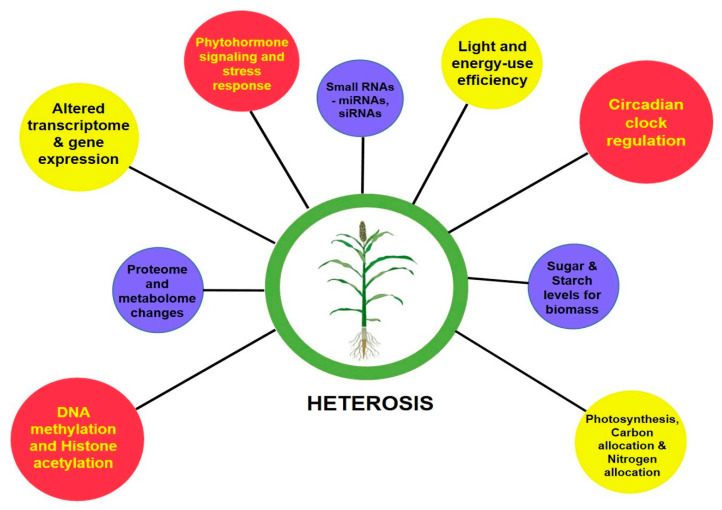
Molecular changes involved in manifesting heterosis.

The perceived greater vigor in the heterotic phenotype than its inbred parental lines is the cumulative result of the coding of genetic information by transcription, translation and their levels of regulation. With the advent of next-generation sequencing (NGS) technologies, genome-wide single-nucleotide polymorphisms (SNPs), insertion or deletions (indels), large structural variations in the form of presence and absence variations (PAVs), and copy number variations (CNVs) that contribute to the phenotypic diversity can be easily deciphered [20,21,22]. To decode the underlying mechanisms determining the degree of vigor differences between inbreds and hybrids, molecular studies were advanced to assess their transcriptome, proteome, epigenome and other regulatory related mechanisms [23]. At a glance, Figure 1 gives a basic idea on the molecular bases of heterosis.

### 2.1. Transcriptomics View on Heterosis 

Hybridization of the inbred parental lines leads to interactions between the nucleus and cytoplasm and the resulting changes at the cellular and molecular level leads to the altered patterns of gene expression. Genome-wide modifications in the gene expression levels and their mechanism of actions in the hybrids vis-à-vis its inbreds have been documented in several hybrids of maize [24,25,26], rice [27], wheat [28], cotton [29], *Arabidopsis* [30,31,32], etc. Transcriptomics and its potential in heterosis can best be viewed as a transitional phase in between the genetic information and the plant phenotype which specifically measures the relative contribution of each allele in a hybrid [33]. To identify the genes involved in heterosis using transcriptome analyses, technologies like microarray, RNA-sequencing (RNA-seq), etc., are being used to compare inbred parental lines with their F_1_ progenies. From the initial transcriptomic studies on several crop species, it was believed that favorable gene expression levels in hybrids are predominant when compared to its inbred parents [34,35,36,37]. However, it is important to note that differential gene expression levels may not directly correspond to the protein activity between inbred parental lines and hybrids, or to the observed heterosis in the hybrids, and post transcription/translation regulations need to be taken into account [14,38]. 

### 2.2. Proteomics View on Heterosis

Studying the role of proteins in determining heterosis is important, as changes at the level of transcripts may not always reflect at the protein level due to various post-transcriptional and translational regulatory mechanisms [38]. The generalized concept is that, in the inbred parental lines protein metabolism is higher due to unstable protein levels which require more energy to quench. Consequently, less energy remains for its vegetative growth, biomass, and yield in the end. The genetic basis for this condition in inbred parental lines is due to the lack of allelic choice in their homozygous condition with only two alleles, whereas hybrids in polyploidy condition will have more alleles and thus display faster growth rates due to increased cell divisions [39].

With the advent of 2-D gel electrophoresis (2-DGE) along with mass spectrometry (MS), numerous studies have been reported to identify the differentially expressed proteins (DEPs) determining heterosis. In addition, MS-based protein detection and quantification depend upon the two isobaric labeling reagents like tandem mass tags (TMT) and isobaric tags for relative and absolute quantification (iTRAQ), which are employed to detect altered proteins or DEPs in heterotic phenotypes [38,40]. To date, the majority of the DEPs determining heterosis have been identified in major crop species like maize, wheat and rice from the tissue samples of the embryo, root, and leaf [41,42,43]. It is apparent from these studies that the majority of the DEPs identified between inbreds and hybrids are due to the non-additive gene effects, and it has also been reported that these DEPs belong to the pathways of signal transduction, glycolysis, photosynthesis, disease resistance, carbon metabolism, protein, amino acid metabolism, etc. [44]. These results indicate that the degree of heterosis was dependent upon the incidence of protein isoforms or modifications [23].

### 2.3. Epigenomics View on Heterosis

DNA methylation: The resulting vigor in hybrids after the hybridization of two distant inbred parental lines is often linked with epigenetic modifications, viz., DNA methylation [45], chromatin structure modification [46], histone acetylation [47], or small RNA induced regulations, etc. [48]. Genome activity and the cellular development of any crop species are indeed regulated by DNA methylation. In most plant species, DNA methylation takes place on the 5’ position of cytosine residues at CG, CHH and CHG regions (where H may be A, T or C) by DNA methyltransferases [49,50]. The degree of DNA methylation change in the hybrids depends on the diversity among inbred parents [51]. Using bisulfite and siRNA sequencing, differential methylated loci were identified in the rice hybrids (Nipponbare and Indica) and their parents to decipher the role of DNA methylation in causing epigenetic heritability [52]. In the allopolyploids of *Arabidopsis*, genes with cis- but not trans-regulatory changes were augmented in loci that were hypo-methylated or hyper-methylated [53]. The manifestation of heterosis by DNA methylation is mainly mediated by the suppression of the transcription process of the regulatory genes involved in enhancing inbreeding depression or by promoting the expression of genes for heterosis [54]. Greaves et al. [55] suggested that DNA methylation sites in hybrids were frequently associated with regions that are differentially methylated in their inbred parents. In particular, methylated regions in the inbred parents were usually covered by the siRNA levels, and this implies that RNA-directed DNA methylation (RdDM) pathway may induce the remodeling of DNA methylation sites in hybrids to manifest heterosis [56].

Apart from DNA methylation, another epigenetic system involved in hybrid vigor is histone modification. These modifications can occur at the post-translational level for the amino acids in histone proteins at the N-terminal tails in the form of methylation, phosphorylation, and acetylation of certain residues [57]. Usual modifications will occur with the histones like H3K9ac and H3K4me3 found in euchromatin regions with active gene expression. Meanwhile, histones H3K27me3 and H3K4me3 were found in the pericentromeric, heterochromatin and transposable element (TE) regions with low transcript levels [58]. He et al. [27] compared differential expression patterns of H3K4me3 and H3K27me3 between hybrids and parents of rice subspecies and found that H3K4me3 is transcriptionally active and H3K27me3 is inactive. In maize F_1_ hybrid endosperm-derived transcriptomes, a histone alternate HTA112 showed significant variations in expression when compared to its parental lines [59]. These studies, though limited, raised the possibility of inducing heterosis through epigenetic histone modifications. 

Small RNAs: Small RNAs, mediating gene expression, and epigenetic regulation refer to the class of microRNAs (miRNAs), small interfering RNAs (siRNAs), and *trans*-acting siRNAs (tasiRNAs) [60]. Usually, siRNAs will maintain the genome stability by affecting the genes tagged with transposable elements (TEs) and also by retaining the stable inheritance of repeat-associated siRNAs, whereas miRNAs and tasiRNAs are involved in controlling morphological and developmental traits [61]. The combination of distant inbred parental siRNAs in the hybrids will exert both *cis* and *trans*-acting effects on TEs and TE-coding genes which causes genomic instability thereby leading to the abortive embryo and endosperm formations termed as hybrid lethality [62]; on the contrary, some small RNAs also play positive regulatory roles in terms of protection from the genomic shock, ultimately leading to hybrid vigor. Small RNA gene expression studies in the hybrids of rice, *Arabidopsis* and maize revealed the downregulated levels of 24-nt siRNAs when compared to their inbred parental lines. The 24-nt siRNAs that are involved in the RdDM mechanism will finally lead to transcriptional repression by stimulating the DNA methylation process. Hence, the reduction of these siRNA levels may lead to the ample expression of protein-coding genes in hybrids [63,64]. The differential expression of siRNAs in hybrids relative to their parents is due to the differences in the promoter regions of small RNA coding genes. Therefore, the epigenetically developed differences at the gene expression level of 24-nt siRNAs in hybrids may contribute to heterosis [7,27,65].

### 2.4. Genes Associated with Heterosis

So far, various potential genes controlling heterosis to some extent have been reported in several crop species. Although heterosis is a complex phenomenon, engineering selective genes may be essential for the rapid and stable induction of heterosis. As organ size controlling genes can be referred to as intrinsic yield-related genes [66,67], in such an attempt, Guo et al. [41] developed transgenics in maize (inbreds and test cross hybrids) by overexpressing *ZAR1* (*Zea mays* ARGOS) gene which is an orthologue of the *Arabidopsis* Auxin Regulated Gene involved in Organ Size (*ARGOS*) [68]. Transgenics showed vigor in terms of yield, organ size and also in resisting drought conditions [41,68]. In maize, the silencing of the Cell Number Regulator 1 gene (*CNR1*) increases the plant and organ size and acts as a direct potential contributor for heterosis [69]. The single dominant flowering controlling gene, SINGLE FLOWER TRUSS (*SFT*) in tomato, which is an orthologue of the *Arabidopsis* FLOWERING LOCUS T (*FT*) gene, is the genetic determinator for the production of flowering hormone florigen, and its loss-of-function mutations showed significant effects in enhancing yield in tomato [70]. In *Arabidopsis*, overexpression of the heterosis-associated AP2/EREBP (APETALA 2/ethylene responsive element binding protein) transcription factor coding gene from Larix *LaAP2L1* results in cell proliferation and in enhanced heterotic traits [71]. Epigenetic modifications of the *Arabidopsis* circadian clock genes, viz., CIRCADIAN CLOCK ASSOCIATED 1 (*CCA1*) and LATE ELONGATED HYPOCOTYL (*LHY*), and the reciprocal regulators, TIMING OF CAB EXPRESSION 1 (*TOC1*) and GIGANTEA (*GI*) facilitated changes at transcriptional level leading to increased vigor in plant development and biomass [51]. In a two-line rice hybrid, Liang-you-pei 9 (LYP9), using the integrated analysis of various ‘omics’ approach two photoperiod sensitive genes RH8 and Ghd7 were identified as being responsible for the plant height and grain yield, leading to heterosis [72]. Genes related to pathways that are positively correlated with manifesting heterosis are DNA replication, repair, plant hormone signal transduction, etc., whereas translation, protein degradation, carbohydrate metabolism, lipid synthesis, and energy metabolism are negatively correlated. However, genes related to transcription, amino acid synthesis, and plant defense were correlated both positively and negatively with heterosis [73]. 

Despite the contribution of several genetic and molecular theories of heterosis in crop plants, yeast (*Saccharomyces cerevisiae*) has provided some valuable insights into our understanding of heterosis. Yeast is a eukaryotic organism that usually multiplies by an asexual mode of reproduction, and isolates of such organisms can be considered as distantly related inbred populations. Thus, studying different combinations/theories of crop heterosis that have been postulated to date in yeast will potentially yield insights to better understand the genetic basis of heterosis and offer a wide scope for applying the acquired strategies to enhance food production [74]. The reciprocal-hemizygosity analysis was employed by Steinmetz et al. [75] to explore the architecture of a quantitative trait locus (QTL) which contributes to heterosis in yeast. Analysis results in the mapping of three tightly linked quantitative genes (*MKT1*, *END3*, and *RHO2*) that are *in cis* and *trans* linkages and their corresponding alleles in heterozygote form exhibit heterosis when compared to the homozygotes. 

Apart from the above reports, which mainly rely on the genes and their networks, metabolic control theory (MCT) attempts to explain heterosis involving metabolic fluxes for the genotype-phenotype (GP) relationship. In this theory, Fievet et al. [76] draw a relationship between heterosis and the above-mentioned theories using a comparison between enzyme-flux and GP relationships. Genetic variability between the parents and hybrids was measured using the enzyme fluxes of glucose, glycerol and acetaldehyde in yeast. For most hybrids, positive heterosis was observed when their metabolic fluxes were compared with the parental fluxes and concluded that heterosis can be manifested to a greater extent provided the parents used for crossing are phenotypically close with wide variations in enzyme concentrations; therefore, using metabolic control analysis, heterosis can be easily exploited by deriving the shape of curves representing phenotype to genotype relationship [76,77]. 

Conclusively, these molecular clues in terms of related pathways and candidate genes for heterosis might be prospective targets, and by combining the above-described ‘omics’ approaches, engineering heterosis-associated genes in nutri-rich cereal like pearl millet could be highly remunerative and rewarding.

## 3. Unifying Theory for Heterosis

Currently, the emerging theory for multi-genic heterosis relies on the efficiency of energy use and protein metabolism. This theory, in short, termed as energy-use efficiency for manifesting multi-genic heterosis, was proposed by Goff [39]. The biology of plants tuned to take light energy to synthesize and assimilate various metabolic compounds and will store in the form of biomass. Based on the above proposed energy-to-biomass model, one can make a clear-cut difference in the energy-consuming process between the hybrids and the inbreds. This model indicates that vigor in the hybrids is due to the substantial reduction in the energy-consuming process during protein metabolism when compared to its inbred counterparts. This metabolic adaptation in hybrids helps to conserve energy from basic pathways and use it for higher growth rates and biomass. This type of energy-use efficiency was reported in *Brassica napus* hybrids, and accounted for a 5% increase in yield compared to the inbreds [78]. By remodeling the circadian clock to a higher magnitude, Ni et al. [79] further tested this model, which resulted in increased photosynthetic efficiency for increased biomass and hybrid vigor. Hybrids of *Arabidopsis* during the early developmental stages performed higher metabolic activity and better energy-use efficiency than the inbreds [80]. It is becoming apparent that positive modulation of energy in any biological system can be rendered into vigor and biomass.

## 4. Exploitation of Heterosis in Cereals

**Figure 2:**
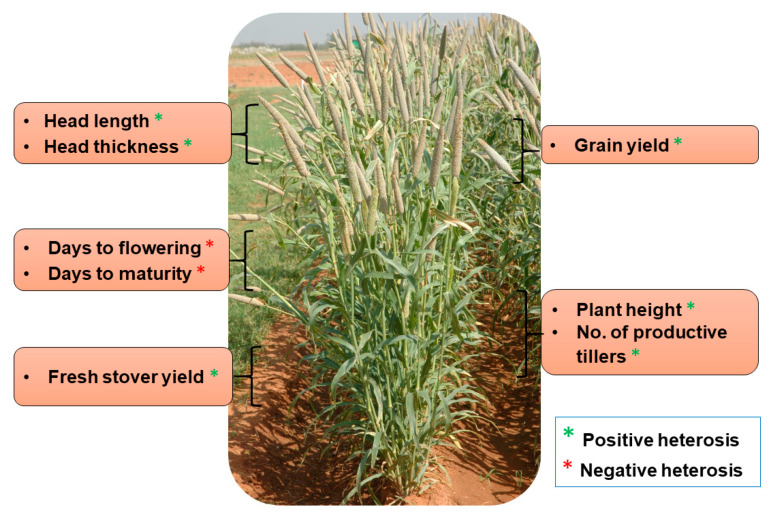
Important agronomic traits exhibiting heterosis in HHB 67 Improved pearl millet hybrid.

The exploitation of heterosis in agriculture (including in both farm animals and crop plants) has been considered a breakthrough, in turn leading to the quantum jump in crop production worldwide. The current grain yield of cereals represents an almost five-fold increase when compared with the yield during the pre-hybrid period. Maize (*Zea mays*) has huge potential for the manifestation of heterosis and its effective exploitation. The number of hybrids in maize is far higher than any other varietal type, as it is gifted with substantial amounts of heterosis for yield and other important agronomic traits [81]. It has been observed that inbred lines of maize exhibit a significant decline in kernel yield and vigor, while those hybrids of two inbred genotypes instantly and entirely recovered [82]. Maize cultivation depends solely on the use of hybrid seeds in maize-growing countries, in which hybrids cover ~70% of the total area under maize cultivation during the 1990–2000 period. Hybrid seed cultivation contributed to a quadrupling of maize kernel yield [82]. Similarly, heterosis is the foundation for the great success of hybrid rice. About 55% of the total area of paddy cultivation accounts for hybrid rice and subsequent increase of rice production by 20 million metric tons in far East Asia, where rice is the major staple food for 70 million people. Hybrid rice varieties have a higher yield advantage of about 10–20% than the conventional inbred varieties [81]. About 73% heterosis, 59% heterobeltiosis and 34% standard heterosis for rice yield were reported in a study performed at the International Rice Research Institute (IRRI) during 1980 and 1981. The genetic basis of heterosis has been exploited in an “immortalized F_2_” population of elite *indica* rice hybrid Shanyou 63; and the results suggest that over-dominance is the basis for heterosis, exhibited in the form of more tiller numbers, grain weight and, grain yield [6]. Apart from these two cereals, where heterosis has been exploited at a large scale, pearl millet is another lesser-known cereal where impressive advances have been made in exploiting heterosis for yield and other quantitative traits through the introduction of first single-cross hybrid in mid-1960s. HHB 67 Improved has been one of the most successful examples of hybrid breeding in pearl millet where substantial heterosis was realized for grain yield and earliness (Table 1, Figure 2). This hybrid is grown over more than 850,000 ha (~10% of the total pearl millet area) in India. The following sections focus on the advances and prospects in various fronts related to heterosis for pearl millet.

## 5. Pearl Millet Introduction and Importance

Pearl millet (*Pennisetum glaucum* (L) R. Br., syn. *Cenchrus americanus* (L.) Morrone) is one of the main staple food crops and sources of fodder and fuel in arid and semi-arid regions of South Asia, India and parts of sub-Saharan Africa. It is gaining importance as climate-smart nutri cereal, and is one of the most extensively cultivated millets globally [83]. The utility of pearl millet varies, ranging from food and feed, through forage, fodder, building material, to brewing and biofuel, making this an important crop species for food and livelihood security for more than 500 million poor and nutritionally insecure people around the world [84]. It is a small-grained, highly cross-pollinated (due to its protogyny) C_4_ field crop with excellent photosynthetic behavior, suitable for smallholder farming systems, and it belongs to the family *Poaceae* (Grass) and the subfamily *Panicoideae*. Pearl millet was first domesticated in West Africa at the southern edge of the Sahara during in approximately 2500 BC [85]. Pearl millet is cultivated on ~27 million hectares and is the main staple diet for more than 90 million resource-poor and marginal farmers around the world. Its short life cycle, high tillering, low input requirement, and high biomass production capacity make this crop highly desirable for marginal farmers. Pearl millet fodder plays an important role in the sustainability of low-input livestock systems. The ever-increasing demand for meat and milk is reflected in the increasing price of pearl millet and other straw cereals [86]. Starch, processed food and alcohol industries also require pearl millet in huge quantities [87].

### 5.1. Climate Resilience

Being a C_4_ crop with climate-resilient attributes allows pearl millet to grow not only in the harshest conditions including soils with low moisture, high pH, high salinity, low fertility and high Al^3+^ saturation, but also in regions prone to frequent drought with low rainfall (annual average rainfall <250 mm) and high temperature, where other cereals fail to survive and produce grain [88,89,90]. Another major potential of this crop is to tolerate air temperatures greater than 42 °C during the reproductive phase, which means crops can be cultivated by irrigation during the very hot summers of north-western parts of India [91]. 

### 5.2. Nutritional Aspects

Pearl millet grain is highly nutritious, which is evident from its high contents of proteins (8–60%), fats (3–4.6%), vitamins (A and B), carbohydrates (60–78%), and lysine (40% more than maize) [84]. It has low contents of starch, is high in fiber (1.2 g per 100g), and has higher micronutrient (iron and zinc) concentrations than rice, wheat, maize, and sorghum [92,93]. Pearl millet is reported to have higher biological value for grain protein than wheat, and in addition, it is gluten-free and low on tannins. Pearl millet also contains higher energy and protein, even having more balanced amino acid profiles than maize or sorghum [93,94]. Feeding trials of pearl millet in India have also established that its nutritional profiles are even higher than rice and maize [95]. Moreover, it is rich in nutrients, and is a cheaper source of protein, energy, iron, and zinc than other cereals. These attributes have led to pearl millet being the main source of protein, iron (35–50%), and zinc (20–30%) intake for low-income consumers [96]. Along with human consumption, it is also a very good source of nutrition for dairy and poultry as animal feed, and it also has high export demand. It has been reported that pearl millet grain is an efficient alternative to maize and can be used in poultry without compromising chick weight gain or feed efficiency [97]. The recent advancement in pearl millet genetic and genomic resources, along with efficient exploitation of heterosis by utilizing the protogynous flowering nature of this crop for grain and fodder yield, offers enormous opportunities for crop improvement programs to accelerate the rate of genetic gains.

## 6. History of Hybrid Development in Pearl Millet

Pearl millet has a rich genetic diversity for agronomic and adaptation-related traits. Globally enormous efforts have been made on pearl millet genetic improvement to address numerous abiotic and biotic challenges [98,99,100]. This has resulted in the development of several open-pollinated varieties (OPV) and single-cross F_1_ hybrids. The main objective of hybrid breeding is to increase the yield and yield stability by exploiting heterosis. In pearl millet, hybrid development was initiated after the discovery of cytoplasmic sterile lines viz., Tift 23A and Tift 18A and released as male sterile lines [98,101,102]. This identification marked the foundation of hybrid breeding and to increase the yield potentials of hybrids by 25–30% and for the resistance to abiotic and biotic stress over the OPVs [99,100]. In India, the first pearl millet hybrid HB1 was released in 1965 by PAU, Ludhiana. Later, in the phase between the 1970s and 1980s, greater efforts have been made on the genetic diversification of cytoplasm and nuclear genome of parental lines of hybrids; and can be considered as an alternative approach for the development of hybrids towards enhancement of yield and yield stability under various stresses [103]. The identification and use of male sterile lines in the hybrid breeding programs and their commercialization have proven to be one of the major milestones over the 50-year history in the pearl millet crop improvement.

### 6.1. Development of Male Sterile Lines

The development of male sterile lines is an outright necessity for hybrid seed production and exploitation of heterosis. In the early 1950s, pearl millet cultivar development from the landraces through direct selection resulted in limited success due to its narrow genetic variability [104]. Thus, the research focus was shifted towards heterosis breeding by developing male sterile lines, which are characterized by the plant’s failure to produce functional pollen with normal stigma. The phenomenon of male sterility has always been of special interest to plant breeders, as it helps to eliminate laborious manual emasculation in order to produce hybrid seed efficiently and economically [100]. In 1763, male sterility in plants was first observed by German botanist Joseph Gottlieb, and has since been reported in 610 plant species [105]. Reproductive biology in male plants includes a series of events from the stamen meristem to pollination, in which defectiveness in any of these processes can lead to male sterility. These defects are caused by either nuclear or cytoplasmic or mitochondrial genes [106]. 

Based on the inheritance, two types of male sterility have been identified in plants: cytoplasmic, and genic/nucleus-dependent male sterility [105]. Genetic male sterility (GMS) is common in flowering plants and is caused by nuclear genes, in which two lines are required for hybrid seed production, viz., male-sterile line and restorer line (any fertile line can be used as a restorer line), and there is no need for maintainer line. GMS has been identified in major food crops (rice, maize, soya bean, etc.) [105]. However, in pearl millet, very little information is available on GMS that is not well characterized [107,108,109]. In pearl millet, genic male sterility variations have been studied in two male sterile lines (Vg 272 and IP 482), and expression variation in sterility has been observed after crossing with different non-isogenic and isogenic lines, and this variation was attributed to an alteration in the *ms_2_* allele [107]. Because of unstable sterility expression, and an occurrence of male fertile plants of 50%, GMS has not been widely used in pearl millet hybrid breeding.

Cytoplasmic male sterility (CMS) has been well investigated in pearl millet, and is still used in hybrid seed production [100]. CMS is a maternally inherited trait that fails to produce functional pollen due to effects caused by certain nuclear and cytoplasmic genes. This type of sterility is caused by cytoplasm dysfunction, and the progeny of these plants would always be male sterile. CMS cytoplasm is normally identified by the genetic cross or protoplast fusion that separates the cytoplasm from its nuclear restoration of fertility (*Rf*) genes, as these *Rf* genes suppress the CMS system by interacting with the mitochondrial genes led to male sterility [110]. However, in pearl millet, the available CMS systems were developed using genetic crosses, and not protoplast fusion. The three-line breeding system is currently in use for the commercial production of hybrids and requires male sterile (A-), maintainer (B-) and restorer (R-) lines. A-line male sterility is jointly controlled by the interaction of sterility factor and recessive fertility restorer (*rf*) allele [111]. 

### 6.2. Development of CMS System—A_1_, A_2_, A_4_ and A_5_ Systems

CMS is a natural biological phenomenon that has been extensively utilized to improve the forage and grain yield, as made possible by the discovery of CMS cytoplasm lines. Various sources of male-sterile cytoplasm in pearl millet have been reported [102]. These systems can be differentiated by differential expression of male fertility restoration genes or by variations in mitochondrial gene expression. Detailed restriction fragment length polymorphism (RFLP) analysis in pearl millet revealed that there were five CMS cytoplasm that were distinct from one another due to the rearrangement of mitochondrial genes, viz., *cox1* and *atp6,* and *cox3*, in cluster regions, in which *cox1* gene rearrangement led to the formation of A_5_ A_egp_, A_1_ and A_4_ and other gene alterations led to the formation of A_4_ CMS system [112]. Based on the phenotype of the restorer, genes for different forms of CMS have been identified on different linkage groups [98,113].

In pearl millet, the discovery of the commercially and economically viable A_1_ CMS system was first reported in inbred 556 in 1958, and its utilization led to the first-released male sterile line, Tift 23 A_1_ [89]. About a decade after the discovery of the A_1_ CMS system, the first CMS-based forage hybrid Gahi-3 (Tift 23DA1 × Tift 186) was released in the year 1972 [99,114]. Consequently, after incorporating the dwarfing gene in the line Tift 23DA1, TifLeaf 1 was bred [115]. This gave way to TifLeaf 2 by using Tift 85D2A1 as a seed parent [116]. Later, using the Tift8593 seed parent, a high-quality, semi-dwarf profusely leafy pearl millet forage hybrid TifLeaf 3 [117] was released in the USA. Two successful new-generation commercial grain hybrids HGM100 and TifGrain 102 were developed by the Georgia program [118,119,120]. These hybrids were extensively planted and exhibited good yields with higher grain productivity [99].

In India, CMS line Tift 23A_1_ was first introduced in 1969 and was extensively used for the hybrid breeding program [121]. Remarkably, since late 1982, the A_1_ CMS source has enhanced the pearl millet forage and grain yield by 50%, and it occupies approximately 40% of the total pearl millet cultivated area in India. After nearly four decades of hybrid cultivation, most of the released hybrids are based on the A_1_ CMS source. However, over a period of time, the A_1_ hybrid system has exhibited issues like the presence of pollen shedders in the A-lines, yield plateauing, and susceptibility to various pathogens. Thus, the research focus has shifted to the identification of alternative CMS sources in hybrid breeding to overcome pathogen infections. 

In India, two alternative CMS sources were identified in 66A and 67A genetic stocks at PAU, and these were later named the A_2_ and A_3_ CMS sources [122]. Subsequently, several other CMS sources have been reported in different genetic stocks at different centers. A new CMS source called Gero pearl millet was discovered in Ibadan Nigeria [123]. In another study, new sources (PT 732A) and A_5_, A_egp_ were found from ICRISAT genetic sources and gene pools [124,125]. Furthermore, Marchais and Pernes [126] reported a new source (ex-Bornu) that is different from existing sources, which was found from a cross between the wild relative of pearl millet [*Pennisetum violaceum* (Lam.) L. Rich = *P. americanum* ssp. monodii] and a landrace (Tiotande) from Senegal and A_4_ [127]. However, A_1_, A_4_, A_5_ are widely used in the three-line breeding program for the production of hybrid seeds. Nowadays, hybrid development has led away from the A_1_ CMS source, but interestingly, the pearl millet bio-fortification program used both A_1_ and A_4_ CMS sources to release different hybrid varieties [128].

### 6.3. Work Done in India on Pearl Millet Hybrid Breeding

The first set of male-sterile lines, Tift 23A and Tift 18A, were developed in the early 1960s at Tifton, and these lines were widely used in the breeding programs at PAU and Indian Agricultural Research Institute (IARI), laying the foundation of pearl millet hybrid breeding in India. Yadav and Rai [100] reported that pearl millet hybrid development happened in three phases: I, II and III. In brief, in the first phase (1960–1980), the A_1_ cytoplasm of Tift 23A_1_, was widely utilized for hybrid seed production because of its short height, abundant tillering, uniform flowering, and good combining ability. In the first wave of hybrid breeding, the initially released hybrid was Hybrid Bajra 1 (HB 1) in 1965 [123], followed by HB 2, HB 3, HB 4, and HB 5. Of these HB series of hybrids, HB 3 become popular and widespread around the world, and was characterized by bold grain, shorter maturity, and tolerance to drought stress. The cultivation of these hybrids resulted in a nearly 75–100% grain yield increase with respect to existing varieties and enhanced production from 3.5–8.0 t in a span of five years. However, several of these hybrids succumbed to the downy mildew (DM) disease. 

The second phase (1981–1996) mainly emphasized the genetic diversification of hybrid parents and also concentrated efforts on the development of disease-resistance lines along with yield enhancement from the support of ICRISAT and All India Coordinated Millets Improvement Program (AICMIP) [129], which resulted in diverse genetic male sterile lines and an added advantage for hybrid seed production. During this phase, there was a 50% productivity increase over the previous phase, and it was also noted that most of the released hybrids were resistant to DM for various CMS sources and were subsequently utilized instead of the A_1_ source. The third phase (1996–2014) had the long-term objective of genetic diversification of both seed and pollinator parents in which grain productivity was further increased by 24 kg ha^−1^ year^−1^ [130]. This phase also played an important role in the improvement of DM resistance through marker-assisted breeding. 

In 1975, the first wave of downy mildew-resistant hybrids, viz., PHB 10 and PHB 14, were released at PAU [131]. Then, during the years 1972–1988, eight hybrids—BJ 104, BK 560, BD 111, BD 763, Co H2, HHB 45, GHB 27, and GHB 32—were released at IARI. Of these eight, two hybrids (BJ 104 and BK 560) were widely cultivated in India [132]. In the late 1970s, the intensive effort of ICRISAT towards the sustainable improvements of hybrids resulted in two major high-yielding hybrids, ICMH 451 and lCMH 501, which were superior to the check hybrid MBH 110 previously used in AICMIP trials over the years [133]. Furthermore, high-yielding hybrid HHB 67 was released in 1990 by CCS Haryana Agricultural University (HAU) with the support of AICMIP, and this became the most widely used cultivated hybrid in Haryana and Rajasthan [129]. In 2005, HHB 67 Improved was the first product of marker-assisted selection (among all crops) released in India. To address widespread problems of micronutrient malnutrition, ICRISAT developed high-iron bio-fortified hybrids named ICMH 1202, ICMH 1203 and ICMH 1301 [128]. In India, a total of 138 hybrids were released by both the public and private sectors from 1958 to 2014 [129].

In India, apart from the public system, robust pearl millet hybrid breeding exists in the private sector, as well. The latter has played a significant role in the development of high-yielding disease-resistant heterotic hybrids. These private companies produce their proprietary hybrids with yield and consumer preference-related traits [128]. Moreover, the private sector utilizes ICRISAT-bred component lines (CMS, maintainer, and restorer lines) for hybrid seed production. Remarkably, in India, the pearl millet hybrid program mostly depends on the components developed by ICRISAT and AICMIP. Component lines are essential tools, and have played a significant role in hybrid developments with enhanced grain yield, quality and resistance to biotic and abiotic stresses. Strong and successful pearl millet hybrid breeding has always depended on the identification and development of diversified component lines exploiting CMS sources. However, the development of component lines has always been a great challenge for pearl millet breeders in terms of achieving sustainable development of hybrids. 

Recently, the Hybrid Parents Research Consortium (HPRC) platform, created and coordinated by ICRISAT, has contributed greatly to the supply of hybrid parental lines to partners in both the public and private sectors, globally. One good example is that of the ICMH 1201 hybrid, which was developed after 48 field trial evaluations during 2011–2013, along with another eight bio-fortified hybrids developed at ICRISAT with the help of AICMIP. ICMH 1201 had a 38% higher grain yield than the previously developed ICTP 8203 hybrid and exhibited 75 mg kg^−1^ Fe density [128]. 

### 6.4. Development of Restorers and Maintainers

The development of restorer and maintainer lines is essential in a three-line breeding program for the production of hybrid seeds. The maintainer (B-) line is the counterpart of the A-line and is used for A-line maintenance. The B-line is characterized by functional pollens and normal anther. The major difference between the A- and B-lines is a cytoplasmic factor/male sterility causing gene(s) (*ms* genes). The restorer line (R-), which carries the dominant *Rf* gene(s), is used as a male parent and is characterized by normal anthers, functional pollens, and strong fertility restoring ability. It is generally taller than the A-line, with profuse pollen load, good combining ability, and is genetically different from the A-line [133]. *Rf* gene(s) can restore fertility in the male sterile line by inducing cytoplasm and allow the production of fertile seeds in a hybrid plant. Any cultivated line (inbred line and open-pollinated line) can be used as the R-line, but usually, a limited percentage of existing lines carry the *Rf* gene. A CMS-based three-line breeding system greatly enhances the yields in hybrids, but has one major difficulty, which is maintaining the B-line in parallel by ensuring nucleus substitution of the original CMS line with the B-lines through continuous backcrossing.

Different sources of CMS (A_1_–A_5_) are available these have distinguished themselves in pearl millet, having played a significant role in restorer line development. Among these, the A_1_ CMS source has been widely utilized for the development of hybrids because of its higher extent of R-line availability [90]. The utility of other CMS sources in hybrid production has been very poor, due to the unavailability of suitable restorer lines. Several studies have reported that, among the various existing CMS sources, A_4_ and A_5_ have been found to be highly stable, along with the A_1_ source. However, suitable restorers are very poorly available in these sources. Hence, for the development of suitable restorer lines, three different CMS A_1_, A_4_ and A_5_ sources have been used. In one study, moderate to incomplete fertility restoration in both A_4_ and A_5_ CMS sources was clearly observed. A_4_ cytoplasm had higher (16% to 52%) fertility restoration than the A_5_ (20%) cytoplasm [134]. 

Detailed studies on CMS/*Rf* systems have provided clues towards the genetic and molecular mechanism of CMS/*Rf* interactions in 13 plant species (maize, rice and wheat, etc.) based on 28 CMS types. This was possible as a result of the sequencing of the mitochondrial genomes of these crops [110]. Candidate genes for CMS have been identified in which notable observations are the differences in mitochondrial gene arrangements/transcriptome/proteome in CMS lines with the presence or absence of *Rf* genes. Overall, it was noticed that the identified candidate genes were mostly related to mitochondrial electron transport pathways [105]. In the same way, nine *Rf* genes have been identified for fertility restoration in seven plant species, including maize, rice, sorghum, radish, sugar beet, brassica, and petunia. 

In pearl millet, some studies have shown the molecular mechanism of inheritance in male sterility and fertility restoration of CMS systems [102,108,109,113]. However, compared to other cereals, there is a gap related to candidate gene identification and its detailed molecular mechanisms for the various forms of CMS systems. Burton and Athwal [102] reported a single recessive and dominant allele, which are responsible for male sterility and fertility restoration, respectively. Further studies revealed that two major dominant complementary alleles at any one duplicate complementary locus led to male fertility restoration in the A_1_ cytoplasm. Additional studies on male fertility restoration in A_1_ and A_5_ cytoplasm reported that dominant alleles with three loci were responsible, in which at least two complementary loci controlled fertility restoration of these CMS systems [108]. Recently, Pucher et al. [113] used the GBS-based linkage map for the identification of genomic loci for male fertility restoration in A_4_ CMS system with developed KASP markers through desired haplo-block screening. QTL for fertility restoration was observed on linkage group 2 (LG2), in which fertility restoration of this system has arisen as a single copy of the defective gene (monogenic dominant pattern). 

## 7. Heterosis and Genomics

With the advancement in high-throughput genotyping and molecular technologies, the whole genomes of several crop plants and their hybrid combinations have been sequenced with whole-genome transcriptome analyses to classify differential gene expression patterns. Whole-genome sequencing data was further used to develop genomic resources (i.e., DNA markers, genome maps, sequence information) to carry out genetic studies or marker-aided breeding in crop plants [135]. To accelerate the application of genomics to improve yield and quality, scientists generated and analyzed draft genome sequences for many staple crops including rice [136], wheat [137], chickpea [138], pea [139], pigeon pea [140], sorghum [141], etc. Recently, Varshney and his team reported the ~1.79 Gb draft whole-genome sequence of the reference pearl millet line Tift 23D2B1-P1-P5, with an estimated 38,579 genes [90]. Resequencing data was used to carry out the genomic selection to predict grain yield for test crosses in pearl millet. Previously, whole-genome transcriptome analysis was exercised in established inbred parental genotypes and their hybrids to study differential gene expression patterns in terms of gene action to correlate changes in yield gains in crops. Differential gene expression was recorded in maize [142,143], rice [144], wheat [145], sorghum [146], finger millet [147], chickpea [148], and pearl millet [149]. 

Generally, as per transcriptomic analyses for heterosis-based studies, the patterns of gene action have been categorized into additive, dominance, and over-dominance classes. The additive mode of gene action characterizes mid-parental expression within hybrids, whereas the dominance mode represents both low- and high-value parent-like expression. In the over-dominance and under-dominance modes of gene action, the degree of expression of differentially expressed genes in the hybrid is either higher than the high parent value (HPV) or lower than the low parent value (LPV), respectively. The preliminary studies for transcriptome analyses exhibited heterosis as a consequence of best-suited gene expressions in cross-fertilized genotypes or hybrids in comparison to their parental lines [36]. Over the past two to three decades, several types of DNA markers and genomic resources have been developed and deployed in various genetic and breeding studies in pearl millet and many other crop plants [150]. With the advent of advanced next-generation sequencing technology, abundant single-nucleotide polymorphism (SNPs) markers in the genome have been developed in plants and the animal kingdom. These potential molecular markers have been harnessed in different aspects of pearl millet genetic analyses, including genetic diversity [151], linkage mapping (QTL) [152], marker-assisted selection (MAS) [153], genome-wide association studies (GWAS) [154,155], and genomic selection (GS) [156]. Until now, several types of genomic tools, such as restriction fragment length polymorphism (RFLP) [157], amplified fragment length polymorphism (AFLP) [158], random amplified polymorphic DNA (RAPD), expressed sequence tag-derived simple sequence repeats (EST-SSRs) [159], sequence-tagged sites (STSs) [160], genomic simple sequence repeats (gSSRs) [161], Diversity arrays technology (DArT) [162], conserved-intron specific primers (CISP) [163], and single nucleotide polymorphism (SNP) markers [156], have been exploited to accelerate the pace of hybrid breeding programs of pearl millet and other cereal crops [164]. 

QTL-linked markers have been used in a couple of studies in cereals for predicting hybrid performance. Transcriptome/proteome analysis has been used in the study of differential gene expression, which exhibits additive, dominance, and over-dominance effects and in the prediction of hybrid performance. However, the results were influenced by several factors, such as type of tissue, and analytical approaches used in the study. Markers linked to heterosis loci (HLs) other than the QTLs for yield-related traits are used to predict hybrid performance more efficiently than the other marker types. The categorization of germplasm resources into distinct heterotic groups is an underlying exercise for optimum deployment of heterosis in hybrid development [165]. Expressed sequence tag (EST) and genomic SSR markers have been used to classify hybrid parental lines and for the identification of heterotic gene pools in pearl millet [83].

## 8. Development of Heterotic Gene Pools

Effective deployment of hybrids in India led to the remarkable improvement in pearl millet productivity from 305 kg ha^−1^ in the 1950s to the present production of 1132 kg ha^−1^ [91]. However, a narrow genetic base is the predominant constraint for hybrid breeding programs. To address this, germplasm collection, systematic assessment of genetic diversity (by phenotyping and/or genotyping) to develop heterotic groups is needed in order to develop high yielding hybrids with standard heterosis [83]. The fundamental principle for the exploitation of heterosis is to characterize the germplasm into different heterotic groups, which helps breeders to develop inbred lines and also use the available germplasm more efficiently for maximizing hybrid breeding outcomes [165,166]. Heterosis prediction and performance of F_1_ from the hybrid parental generation improve the efficacy of hybrid or improved cultivars by reducing the crossing and field evaluation costs for selecting heterotic crosses [167]. In cross-pollinated crops, the embodiment of wide genetic variability (genetic base) in heterotic pools and schemes for improving combining ability is pivotal for constant genetic gain and must be an integral part of hybrid breeding schemes. Heterotic grouping, along with clusters (patterns), facilitates accurate selection of parents to perform a large number of hybrid combinations within a short time, and also allows us to evade the advancement and evaluation of redundant hybrids.

In pearl millet, efforts are being made by ICRISAT and other public and private sectors to develop genetic pools by introducing the hybrid parental lines from African and Asian origin for augmenting the genetic diversity and to keep up the momentum of genetic gains in this important nutri-cereal [168]. Out-crossing behavior and the wide adaptive nature of pearl millet leads to greater levels of diversity [169,170]. In pearl millet, successful heterosis breeding mainly relies on the development of diverse sets of A-, B- and R-line pools distinguished with wide genetic variability [83]. Very limited information was available in pearl millet for the identification of heterotic gene pools by using genomic technologies. Ramya et al. [83] for the first time defined the heterotic gene pools by using molecular markers, such as EST-SSR (72, of which 69 IPES (ICRISAT pearl millet EST stress), 3 ICMP (ICRISAT millet primer) markers and genomic SSR (6) markers. The plant material used for the study comprised of 342 hybrid parental lines, of which 160 B (maintainer) lines and 182 R (restorer) lines along with world reference germplasm Tift 23D2B1-P1-P5 as control. Clear-cut differentiation and grouping of B lines into 9 clusters and R lines into 11 clusters with slight intrusions were reported. Grouping of inbred lines was assigned based on genetic dissimilarity values (with an average marker genetic distance 0.55) revealed a moderate level of variation in the lines. Later, Singh and Gupta [170] also generated heterotic gene pools in pearl millet using 150 hybrid parental lines comprising of 75 each of B- and R-lines (60 each from ICRISAT, Patancheru and 15 each from CCS HAU, Hisar) using 56 polymorphic SSR markers distributed across all the linkage groups resulted in eight clusters. In another study, a set of 150 advanced hybrid parental lines (75 B lines and 75 R lines) were assessed with 56 highly polymorphic SSR markers distributed across all the chromosomes and approximately 75,000 SNP markers were identified in 117 hybrid parents. Genetic diversity analysis based on these markers has been reported to form clear-cut clustering of B and R lines into different groups [170]. Bharadwaj et al. [171] also reported genetic diversity analysis in 95 B and 95 R lines using 40 SSR markers in pearl millet, resulting in two major clusters of B and R lines, with a few exceptions.

Several other studies in pearl millet grouped the germplasm based on genetic distance using molecular markers. Kapadia et al. [172] studied genetic diversity among 18 pearl millet genotypes using 28 SSR markers differentiated into three major clusters. In another study, a set of novel restorer lines (45) with good combining ability, bred in India and Africa were deployed to assess the genetic diversity using 50 SSR markers, which formed 8 distinct clusters by forming groups of accessions with same or similar pedigrees [173]. Singh et al. [103] reported genetic diversity analysis in 20 pearl millet cultivars which are commercially released including hybrids and open-pollinated varieties (OPV) screened with 21 (out of 60) SSR primers resulted in the formation of three different groups. Another study by Sumanth et al. [174] evaluated 42 pearl millet inbred lines comprising of 22 maintainer and 20 restorer lines with seventeen SSR primers to assess the genetic diversity forming different groups. Stich et al. [175] screened a set of 145 pearl millet inbred derived from 122 landraces, with 20 SSR markers and identified genetic diversity for pearl millet inbreds originating from West and Central African countries for different parameters, viz., gene diversity D, alleles count per locus, and group-specific allele count. Significant achievements in developing heterotic gene pools in pearl millet are listed in Table 2.

## 9. Development of Whole Genome Prediction Models

Genomic prediction and/or selection (GP/GS) is a new and promising breeding strategy with huge potential in which genome-wide markers will be used to predict the genomic estimated breeding value (GEBV) to discover and boost the genetic gain from selection and thus speed up the breeding process in crops [176]. Genomic selection was established to be a cost-effective, sustainable alternative to marker-assisted phenotypic selection for important quantitative traits and hastened crop improvement programs in cereals and other inbreeding crops [176,177,178,179,180,181,182]. In this breeding schema, efficient training population design with both genotypic and phenotypic data is a prerequisite. This design predicts the GEBVs (genome-wide marker and phenotype relationship) of testing population with genotypic data by using genome-wide DNA markers which are in linkage disequilibrium (LD) with QTL, and the predicted GEBVs are used for best parental selection for making new crosses [183]. High-quality GEBVs make GS a potential tool for identifying outperforming offspring in the population, thus accelerating breeding efficiency in crops by the gain from the precise selection of parents [176]. The major advantage of the GS breeding schema over traditional MAS is that GEBVs will be predicted by all the available marker data and not restricted to a selected set of markers (linked to genes). This feature allows GS to track minor effect genes along with major effect genes/QTLs, which further circumvent ascertainment bias as well as information loss. In GP, statistical contraction and Bayesian and other different machine learning procedures were used to cover the effects of several thousand genes [183,184]. Another great advantage of GS is that selection decisions can be taken during the off-season, leading to accelerated genetic gain on an annual basis [185]. GS breeding schemes can rapidly improve important and complex traits with low heritability, aside from the fact that it will also considerably reduce line and hybrid development costs [186]. On the other hand, GS can also be used for simple traits having good heritability, rather than for more complex traits, for which accurate GP is a prerequisite. However, the application of GP/GS in plant breeding has its limitations, including the cost of genotyping and lack of clear and well-defined procedures where GP/GS can be applied in breeding programs [186].

Pearl millet offers a fascinating opportunity to apply GP/GS breeding schema in crop improvement programs, as it is widely employed in both hybrids and open-pollinated production systems. Another advantage is that good quality phenotypic data on different quantitative traits are available for pearl millet hybrids as well as inbred B and R lines. Application of GP/GS in pearl millet breeding enables accurate prediction of hybrid performance together with the best possible resource allocation. In ICRISAT, India, efforts are being made to make use of available whole-genome resequencing (WGRS) data of pearl millet inbred germplasm association panel (PMiGAP) lines together with high-quality phenotyping data of different quantitative traits for evaluating the utility of GP/GS for accurate prediction of combinations for hybrid breeding. Varshney et al. [90] applied genomic selection by using WGRS DNA marker data to predict grain yield for test crosses in different situations, i.e., yield performance in control, early and late stresses, and across environments, and found remarkable prediction with high accuracy for yield performance across environments. It was also reported that, by using the available grain yield data of different hybrids and 302,110 SNPs, some interesting findings were revealed. Here, the ridge regression best linear unbiased prediction method (RR-BLUP) model was trained using grain yield data (training population) from 64 pearl millet hybrids screened in five different environments in replicated trials (during 2004–2013). GS through additive and dominance effects predicted about 170 potential hybrid combinations, of which eleven combinations had already been deployed for the production of promising hybrids, while the remaining prospective combinations can be incorporated into hybrid breeding programs for developing pearl millet hybrids with yield advantage [90]. Assessment of predicted hybrid performance (hierarchical clustering) of possible single cross combinations (167,910) revealed two sets of lines exhibiting improved hybrid performance (about 8%) by crossing each other. This study also identified some interesting heterotic gene pools, these hybrids and combinations could be the prospective nucleus for developing high yielding pearl millet hybrids [90]. Liang et al. [156] evaluated a set of ICRISAT developed inbred pearl millet lines by using GP/GS strategy to assess two potential genotyping datasets (15,306 RAD-seq SNPs and 32,463 tGBS SNPs) and four GS schemes through the implementation of RR-BLUP [178]. The capability of GP was assessed by using projected hybrids from RAD-seq and tGBS genotyping datasets for all the phenotypes of the study by 20 random rounds of cross-validation (five-fold) for tested SNP set. The study, using the GP scheme with hybrid data, generated a median range of prediction accuracies for tested phenotypic data: 0.87–0.89 (days to flowering); 0.72–0.73 (plant height); 0.48–0.51 (grain yield) and 0.73–0.74 (1000—grain weight). Hybrid GEBVs can be moderately refined by integrating inbred phenotypic data sets when inbred and hybrid trait values and mean trait values of the test population are related. It has also been reported that, although the historical breeding records a great source of pearl millet inbred phenotypic data, naive integration of historical data in hybrid breeding could lower prediction accuracy. However, by controlling the heterosis per se (inbred genotype and trait data) may improve the accuracy of GEBVs of hybrids in pearl millet [156]. Applicability of the GP/GS model in pearl millet improvement could be expanded by incorporating the hybrids from diverse genetic backgrounds.

## 10. Future Prospects

In current scenarios of ‘climate change’ and burgeoning population, a climate change-ready smart crop like pearl millet offers exciting opportunities towards improving the grain and fodder yield for different agro-ecologies globally. The availability of cutting-edge genomic resources and enabling tools/technologies will aid in the development of precise heterotic gene pools leading to the development of heterotic hybrids translating to greater impacts. It may also help develop robust whole-genome prediction models by incorporating linked markers for both yield stability, as well as yield-enhancing traits such as combining ability, yield and stress tolerance related genomic regions. Development of new breeding technologies (NBTs) such as next-generation speed breeding, integrating genomic prediction models supported by big data, cloud computing, machine learning, and artificial intelligence may lead to the next quantum jump in the yield by accelerating the rate of genetic gains.

## Figures and Tables

**Table 1:** Manifestation of heterosis for different agronomic traits in a popular pearl millet hybrid, HHB 67 Improved.

Traits	Seed Parent	Pollen Parent	F_1_ Hybrid	Heterosis % (Over Better Parent)
Days to flowering	47	47	36	−23.40
Days to maturity	77	72	69	−4.12
Plant height (cm)	110	100	160	45.45
No. of productive tillers	3	4	6	50.00
Head length (cm)	17	10	21	23.53
Head thickness (mm)	21	14	22	4.76
Grain yield (g/plant)	15	8	27	80.00
Fresh stover yield (g/plant)	26	30	97	223.22

**Table 2:** Important studies in pearl millet related to the development of heterotic gene pools and germplasm grouping (genetic diversity analysis).

Study	Germplasm	Markers	Reference
1	150 hybrid parental lines (75 B lines and 75 R lines)	56 polymorphic SSR markers	[166]
2	342 hybrid parental lines (160 B lines and 182 R lines) and world reference germplasm Tift 23D2B1-P1-P5 (control)	EST-SSR (72 of which 69 IPES and 3 ICMP) markers and genomic SSR (6) markers	[83]
3	150 advanced hybrid parental lines (75 B lines and 75 R lines)	56 highly polymorphic SSR markers and approximately 75,000 SNP markers	[170]
4	95 B and 95 R lines	40 SSR markers	[171]
5	18 pearl millet genotypes	28 SSR markers	[172]
6	A set of 45 novel R lines	50 SSR markers	[168]
7	20 pearl millet hybrids and open-pollinated varieties	60 SSR markers	[169]
8	42 inbred lines (22 B lines and 20 R lines)	17 SSR markers	[174]
9	145 pearl millet inbreds derived from 122 landraces	20 SSR markers	[175]

## References

[1] Coors C.G., Pandey S. (1999). The Genetics and Exploitation of Heterosis in Crops.

[2] Shull G.H. (1948). What is heterosis?. Genetics.

[3] Darwin C.R. (1876). The Effects of Cross and Self Fertilization in the Vegetable Kingdom.

[4] Rajendrakumar P., Hariprasanna K., Seetharama N. (2015). Prediction of Heterosis in Crop Plants—Status and Prospects. Am. J. Exp. Agric..

[5] Mendel G. (1977). Experiments in Plant Hybridization.

[6] Zhou G., Chen Y., Yao W., Zhang C., Xie W., Hua J., Xing Y., Xiao J., Zhang Q. (2012). Genetic composition of yield heterosis in an elite rice hybrid. Proc. Natl. Acad. Sci. USA.

[7] Groszmann M., Greaves I.K., Albertyn Z.I., Scofield G.N., Peacock W.J., Dennis E.S. (2011). Changes in 24-nt siRNA levels in Arabidopsis hybrids suggest an epigenetic contribution to hybrid vigor. Proc. Natl. Acad. Sci. USA.

[8] Duvick D.N., Coors J.G., Pandey S. (2015). Heterosis: Feeding People and Protecting Natural Resources. Soil Surv. Land Use Plan..

[9] Just T., Reed H.S., Verdoorn F. (1943). A Short History of the Plant Sciences. Am. Midl. Nat..

[10] Bruce A.B. (1910). The Mendelian Theory of Heredity and the Augmentation of Vigor. Science.

[11] Jones D.F. (1917). Dominance of Linked Factors as a Means of Accounting for Heterosis. Proc. Natl. Acad. Sci. USA.

[12] East E.M. (1936). Heterosis. Genetics.

[13] Chen Z.J. (2010). Molecular mechanisms of polyploidy and hybrid vigor. Trends Plant. Sci..

[14] Fu D., Xiao M., Hayward A., Fu Y., Liu G., Jiang G., Zhang H. (2014). Utilization of crop heterosis: A review. Euphytica.

[15] Charlesworth B., Charlesworth D. (1999). The genetic basis of inbreeding depression. Genet. Res..

[16] McClintock B. (1984). The significance of responses of the genome to challenge. Science.

[17] Ha M., Lu J., Tian L., Ramachandran V., Kasschau K.D., Chapman E.J., Carrington J.C., Chen X., Wang X.-J., Chen Z.J. (2009). Small RNAs serve as a genetic buffer against genomic shock in Arabidopsis interspecific hybrids and allopolyploids. Proc. Natl. Acad. Sci. USA.

[18] Lippman Z.B., Cohen O., Alvarez J.P., Abu-Abied M., Pekker I., Paran I., Eshed Y., Zamir D. (2008). The Making of a Compound Inflorescence in Tomato and Related Nightshades. PLoS Biol..

[19] Velu G., Rai K.N., Muralidharan V., Longvah T., Crossa J. (2011). Gene effects and heterosis for grain iron and zinc density in pearl millet (Pennisetum glaucum (L.) R. Br). Euphytica.

[20] Díaz A., Zikhali M., Turner A.S., Isaac P., Laurie D.A. (2012). Copy number variation affecting the photoperiod-B1 and vernalization-A1 genes is associated with altered flowering time in wheat (Triticum aestivum). PLoS ONE.

[21] Zmienko A., Samelak-Czajka A., Kozlowski P., Figlerowicz M. (2013). Copy number polymorphism in plant genomes. Theor. Appl. Genet..

[22] Saxena R.K., Edwards D., Varshney R.K. (2014). Structural variations in plant genomes. Brief. Funct. Genom..

[23] Kaeppler S. (2012). Heterosis: Many Genes, Many Mechanisms—End the Search for an Undiscovered Unifying Theory. Isrn Bot..

[24] Swanson-Wagner R.A., Jia Y., DeCook R., Borsuk L.A., Nettleton D.S., Schnable P.S. (2006). All possible modes of gene action are observed in a global comparison of gene expression in a maize F1 hybrid and its inbred parents. Proc. Natl. Acad. Sci. USA.

[25] Guo M., Rupe M.A., Yang X., Crasta O., Zinselmeier C., Smith O.S., Bowen B. (2006). Genome-wide transcript analysis of maize hybrids: Allelic additive gene expression and yield heterosis. Theor. Appl. Genet..

[26] Stupar R.M., Springer N.M. (2006). Cis-transcriptional Variation in Maize Inbred Lines B73 and Mo17 Leads to Additive Expression Patterns in the F1Hybrid. Genetics.

[27] He G., Zhu X., Elling A.A., Chen L., Wang X., Guo L., Liang M., He H., Zhang H., Chen F. (2010). Global Epigenetic and Transcriptional Trends among Two Rice Subspecies and Their Reciprocal Hybrids. Plant. Cell.

[28] Wang Z., Ni Z., Wu H., Nie X., Sun Q. (2006). Heterosis in root development and differential gene expression between hybrids and their parental inbreds in wheat (Triticum aestivum L.). Theor. Appl. Genet..

[29] Flagel L., Udall J.A., Nettleton D.S., Wendel J.F. (2008). Duplicate gene expression in allopolyploid Gossypium reveals two temporally distinct phases of expression evolution. BMC Biol..

[30] Shen H., He H., Li J., Chen W., Wang X., Guo L., Peng Z., He G., Zhong S., Qi Y. (2012). Genome-Wide Analysis of DNA Methylation and Gene Expression Changes in Two Arabidopsis Ecotypes and Their Reciprocal Hybrids. Plant. Cell.

[31] Fujimoto R., Taylor J., Shirasawa S., Peacock W.J., Dennis E.S. (2012). Heterosis of Arabidopsis hybrids between C24 and Col is associated with increased photosynthesis capacity. Proc. Natl. Acad. Sci. USA.

[32] (2011). 32. Fujimoto, R; Taylor, J.M; Sasaki, T; Kawanabe, T; Dennis, E.S. Genome wide gene expression in artificially synthesized amphidiploids of Arabidopsis. Plant Mol. Biol..

[33] Schnable P.S., Springer N.M. (2013). Progress Toward Understanding Heterosis in Crop Plants. Annu. Rev. Plant. Biol..

[34] Comings D.E., MacMurray J.P. (2000). Molecular Heterosis: A Review. Mol. Genet. Metab..

[35] Baranwal V.K., Mikkilineni V., Zehr U.B., Tyagi A.K., Kapoor S. (2012). Heterosis: Emerging ideas about hybrid vigour. J. Exp. Bot..

[36] Stupar R.M., Gardiner J., Oldre A., Haun W.J., Chandler V.L., Springer N.M. (2008). Gene expression analyses in maize inbreds and hybrids with varying levels of heterosis. BMC Plant. Biol..

[37] Fujimoto R., Uezono K., Ishikura S., Osabe K., Peacock W.J., Dennis E.S. (2018). Recent research on the mechanism of heterosis is important for crop and vegetable breeding systems. Breed. Sci..

[38] Xing J., Sun Q., Ni Z. (2016). Proteomic patterns associated with heterosis. Biochim. Biophys. Acta (Bba)—Proteins Proteom..

[39] Goff S.A. (2010). A unifying theory for general multigenic heterosis: Energy efficiency, protein metabolism, and implications for molecular breeding. New Phytol..

[40] Wang J., Yu Q., Xiong H., Wang J., Chen S., Yang Z., Dai S. (2016). Proteomic Insight into the Response of Arabidopsis Chloroplasts to Darkness. PLoS ONE.

[41] Guo B., Chen Y., Zhang G., Xing J., Hu Z., Feng W., Yao Y., Peng H., Du J., Zhang Y. (2013). Comparative Proteomic Analysis of Embryos between a Maize Hybrid and Its Parental Lines during Early Stages of Seed Germination. PLoS ONE.

[42] Song X., Ni Z., Yao Y., Xie C., Li Z., Wu H., Zhang Y., Sun Q. (2007). Wheat (Triticum aestivum L.) root proteome and differentially expressed root proteins between hybrid and parents. Proteomics.

[43] Zhang C., Yin Y., Zhang A., Lu Q., Wen X., Zhu Z., Zhang L., Lu C. (2012). Comparative proteomic study reveals dynamic proteome changes between super hybrid rice LYP9 and its parents at different developmental stages. J. Plant. Physiol..

[44] Marcon C., Schutzenmeister A., Schutz W., Madlung J., Piepho H.P., Hochholdinger F. (2010). Non-additive protein accumulation patterns in maize (Zea mays L) hybrids during embryo development. J. Prot. Res..

[45] Parisod C., Salmon A., Zerjal T., Tenaillon M., Grandbastien M.-A., Ainouche M. (2009). Rapid structural and epigenetic reorganization near transposable elements in hybrid and allopolyploid genomes in Spartina. New Phytol..

[46] Moghaddam A.M.B., Colot V., Mette F., Houben A. (2007). Heterosis and chromatin structure: Does intraspecific hybridization trigger epigenetic changes?. Chrom. Res..

[47] Tanabata T., Taguchi-Shiobara F., Kishimoto N., Chechetka S., Shinomura T., Habu Y. (2010). A phenomics approach detected differential epigenetic growth regulation between inbreds and their hybrid in Oryza sativa. Mol. Breed..

[48] Groszmann M., Greaves I.K., Albert N., Fujimoto R., Helliwell C.A., Dennis E.S., Peacock W.J. (2011). Epigenetics in plants—Vernalization and hybrid vigour. Biochim. Biophys. Acta.

[49] Law J.A., Jacobsen S.E. (2010). Establishing, maintaining and modifying DNA methylation patterns in plants and animals. Nat. Rev. Genet..

[50] Fernie A.R., Chen B., Wang X., Li X., Li J., He H., Yang M., Lu L., Qi Y., Wang X. (2013). Conservation and divergence of transcriptomic and epigenomic variation in maize hybrids. Genome Biol..

[51] Chen Z.J. (2013). Genomic and epigenetic insights into themolecular bases of heterosis. Nat. Rev. Genet..

[52] Chodavarapu R.K., Feng S., Ding B., Simon S.A., Lopez D., Jia Y., Wang G.-L., Meyers B.C., Jacobsen S.E., Pellegrini M. (2012). Transcriptome and methylome interactions in rice hybrids. Proc. Natl. Acad. Sci. USA.

[53] Shi X., Ng D.W.-K., Zhang C., Comai L., Ye W., Chen Z.J. (2012). Cis- and trans-regulatory divergence between progenitor species determines gene-expression novelty in Arabidopsis allopolyploids. Nat. Commun..

[54] Nakamura S., Hosaka K. (2009). DNA methylation in diploid inbred lines of potatoes and its possible role in the regulation of heterosis. Theor. Appl. Genet..

[55] Greaves I.K., Groszmann M., Ying H., Taylor J., Peacock W.J., Dennis E.S. (2012). Trans chromosomal methylation in Arabidopsis hybrids. Proc. Natl. Acad. Sci. USA.

[56] Greaves I.K., Eichten S.R., Groszmann M., Wang A., Ying H., Peacock W.J., Dennis E.S. (2016). Twenty-four–nucleotide siRNAs produce heritable trans-chromosomal methylation in F1 Arabidopsis hybrids. Proc. Natl. Acad. Sci. USA.

[57] Berger S.L. (2007). The complex language of chromatin regulation during transcription. Nature.

[58] Roudier F., Teixeira F.K., Colot V. (2009). Chromatin indexing in Arabidopsis: An epigenomic tale of tails and more. Trends Genet..

[59] Jahnke S., Sarholz B., Thiemann A., Kühr V., Gutierrez-Marcos J.F., Geiger H.H., Piepho H.-P., Scholten S. (2009). Heterosis in early seed development: A comparative study of F1 embryo and endosperm tissues 6 days after fertilization. Theor. Appl. Genet..

[60] Chen X. (2009). Small RNAs and their roles in plant development. Annu. Rev. Cell Dev. Biol..

[61] Xie F., He Z., Esguerra M.Q., Qiu F., Ramanathan V. (2013). Determination of heterotic groups for tropical Indica hybrid rice germplasm. Theor. Appl. Genet..

[62] Ng D.W.-K., Lu J., Chen Z.J. (2012). Big roles for small RNAs in polyploidy, hybrid vigor, and hybrid incompatibility. Curr. Opin. Plant. Biol..

[63] Greaves I.K., Gonzalez-Bayon R., Wang L., Zhu A., Liu P.-C., Groszmann M., Peacock W.J., Dennis E.S. (2015). Epigenetic Changes in Hybrids. Plant. Physiol..

[64] Groszmann M., Greaves I.K., Fujimoto R., Peacock W.J., Dennis E.S. (2013). The role of epigenetics in hybrid vigour. Trends Genet..

[65] Barber W.T., Zhang W., Win H., Varala K., Dorweiler J.E., Hudson M.E., Moose S.P. (2012). Repeat associated small RNAs vary among parents and following hybridization in maize. Proc. Natl. Acad. Sci. USA.

[66] Busov V., Brunner A.M., Strauss S.H. (2008). Genes for control of plant stature and form. New Phytol..

[67] Krizek B.A. (2009). Making bigger plants: Key regulators of final organ size. Curr. Opin. Plant. Biol..

[68] Hu Y., Xie Q., Chua N.-H. (2003). The Arabidopsis Auxin-Inducible Gene ARGOS Controls Lateral Organ Size. Plant. Cell.

[69] Guo M., Rupe M.A., Dieter J.A., Zou J., Spielbauer D., Duncan K.E., Howard R.J., Hou Z., Simmons C.R. (2010). Cell Number Regulator1 Affects Plant and Organ Size in Maize: Implications for Crop Yield Enhancement and Heterosis. Plant. Cell.

[70] Krieger U., Lippman Z.B., Zamir D. (2010). The flowering gene SINGLE FLOWER TRUSS drives heterosis for yield in tomato. Nat. Genet..

[71] Li A., Zhou Y., Jin C., Song W., Chen C., Wang C. (2013). LaAP2L1, a Heterosis-Associated AP2/EREBP Transcription Factor of Larix, Increases Organ Size and Final Biomass by Affecting Cell Proliferation in Arabidopsis. Plant. Cell Physiol..

[72] Li D., Huang Z., Song S., Xin Y., Mao D., Lv Q., Zhou M., Tian D., Tang M., Wu Q. (2016). Integrated analysis of phenome, genome, and transcriptome of hybrid rice uncovered multiple heterosis-related loci for yield increase. Proc. Natl. Acad. Sci. USA.

[73] Huang Y., Zhang L., Zhang J., Yuan D., Xu C., Li X., Zhou D.-X., Wang S., Zhang Q. (2006). Heterosis and polymorphisms of gene expression in an elite rice hybrid as revealed by a microarray analysis of 9198 unique ESTs. Plant. Mol. Biol..

[74] Shapira R., Levy T., Shaked S., Fridman E., David L. (2014). Extensive heterosis in growth of yeast hybrids is explained by a combination of genetic models. Heredity.

[75] Steinmetz L.M., Sinha H., Richards D.R., Spiegelman J.I., Oefner P.J., McCusker J.H., Davis R.W. (2002). Dissecting the architecture of a quantitative trait locus in yeast. Nature.

[76] Fiévet J.B., Nidelet T., Dillmann C., De Vienne D. (2018). Heterosis Is a Systemic Property Emerging From Non-linear Genotype-Phenotype Relationships: Evidence From in Vitro Genetics and Computer Simulations. Front. Genet..

[77] Cornish-Bowden A., Cárdenas M.L. (2020). Contrasting theories of life: Historical context, current theories. In search of an ideal theory. Biosystems.

[78] Hauben M., Haesendonckx B., Standaert E., Van Der Kelen K., Azmi A., Akpo H., Van Breusegem F., Guisez Y., Bots M., Lambert B. (2009). Energy use efficiency is characterized by an epigenetic component that can be directed through artificial selection to increase yield. Proc. Natl. Acad. Sci. USA.

[79] Ni Z., Kim E.-D., Ha M., Lackey E., Liu J., Zhang Y., Sun Q., Chen Z.J. (2008). Altered circadian rhythms regulate growth vigour in hybrids and allopolyploids. Nature.

[80] Meyer R.C., Witucka-Wall H., Becher M., Blacha A., Boudichevskaia A., Dörmann P., Fiehn O., Friedel S., Von Korff M., Lisec J. (2012). Heterosis manifestation during early Arabidopsis seedling development is characterized by intermediate gene expression and enhanced metabolic activity in the hybrids. Plant J..

[81] Mulualem T., Abate M. (2016). Heterotic Response in Major Cereals and Vegetable Crops. Int. J. Plant. Breed. Genet..

[82] Shull G.H. (1908). The Composition of a Field of Maize. J. Hered..

[83] Ramya A.R., Ahamed M.L., Satyavathi C.T., Rathore A., Katiyar P., Raj A.G.B., Kumar S., Gupta R., Mahendrakar M., Yadav R. (2018). Towards Defining Heterotic Gene Pools in Pearl Millet [Pennisetum glaucum (L.) R. Br.]. Front. Plant Sci..

[84] Bollam S., Pujarula V., Srivastava R.K., Gupta R.K. (2018). Genomic Approaches to Enhance Stress Tolerance for Productivity Improvements in Pearl Millet. Biotechnologies of Crop Improvement, Volume 3.

[85] Manning K., Pelling R., Higham T., Schwenniger J.-L., Fuller D. (2011). 4500-Year old domesticated pearl millet (Pennisetum glaucum) from the Tilemsi Valley, Mali: New insights into an alternative cereal domestication pathway. J. Archaeol. Sci..

[86] Hash T., Raj A.B., Lindup S., Sharma A., Beniwal C., Folkertsma R., Mahalakshmi V., Zerbini E., Blümmel M. (2003). Opportunities for marker-assisted selection (MAS) to improve the feed quality of crop residues in pearl millet and sorghum. Field Crop. Res..

[87] Basavaraj S.H., Singh V.K., Singh A., Singh A., Singh A., Anand D., Yadav S., Ellur R.K., Singh D., Krisnan S.G. (2010). Marker-assisted improvement of bacterial blight resistance in parental lines of Pusa RH10, a superfine grain aromatic rice hybrid. Mol. Breed..

[88] Nambiar V.S., Dhaduk J.J., Sareen N., Shahu T. (2011). and Desai, R. Potential functional implications of pearl millet (Pennisetum glaucum) in health and disease. J. Appl. Pharm. Sci..

[89] Vadez V., Hash T., Bidinger F.R., Kholova J., Monneveux P., Ribaut J.M. (2014). Phenotyping pearl millet for adaptation to drought. Drought Phenotyping in Crops: From Theory to Practice.

[90] Varshney R.K., Shi C., Thudi M., Mariac C., Wallace J.G., Qi P., Zhang H., Zhao Y., Wang X., Rathore A. (2017). Pearl millet genome sequence provides a resource to improve agronomic traits in arid environments. Nat. Biotechnol..

[91] Gupta S.K., Nepolean T., Sankar S.M., Rathore A., Das R.R., Rai K.N., Hash C.T. (2015). Patterns of Molecular Diversity in Current and Previously Developed Hybrid Parents of Pearl Millet [Pennisetum glaucum (L.) R. Br.]. Am. J. Plant Sci..

[92] Souci S.W., Fachmann W., Kraut H. (2000). Food Composition and Nutrition Tables (No. Ed. 6).

[93] Tako E., Reed S.M., Budiman J., Hart J., Glahn R. (2015). Higher iron pearl millet (Pennisetum glaucum L.) provides more absorbable iron that is limited by increased polyphenolic content. Nutr. J..

[94] Finkelstein J.L., Mehta S., Udipi S.A., Ghugre P.S., Luna S.V., Murray-Kolb L.E., Przybyszewski E.M., Haas J.D. (2015). A randomized trial of iron-biofortified pearl millet in school children in India. J. Nutr..

[95] Lardy G.P., Adams D.C., Klopfenstein T.J., Patterson H.H. (2004). Building beef cow nutritional programs with the 1996 NRC beef cattle requirements model. J. Anim. Sci..

[96] Vadez V., Hash T., Bidinger F.R., Kholova J. (2012). II.1.5 Phenotyping pearl millet for adaptation to drought. Front. Physiol..

[97] Smith R.L., Jensen L.S., Hoveland C.S., Hanna W.W. (1989). Use of Pearl Millet, Sorghum, and Triticale Grain in Broiler Diets. J. Prod. Agric..

[98] Burton G.W. (1907). Cytoplasmic Male-Sterility in Pearl Millet (Pennisetum glaucum) (L.) R. Br.1. Agron. J..

[99] Serba D.D., Perumal R., Tesso T., Min D. (2017). Status of Global Pearl Millet Breeding Programs and the Way Forward. Crop. Sci..

[100] Yadav O.P., Rai K.N. (2013). Genetic Improvement of Pearl Millet in India. Agric. Res..

[101] Burton G.W. (1965). Pearl millet Tift 23A released. Crops Soils.

[102] Burton G.W., Athwal D.S. (1967). Two Additional Sources of Cytoplasmic Male-Sterility in Pearl Millet and Their Relationship to Tift 23A 1. Crop. Sci..

[103] Singh S.P., Satyavathi C.T., Sankar S.M. Diversification of male sterility sources with special reference to biotic stresses. Proceedings of the New paradigms in heterosis breeding: Conventional and molecular approaches, G.B Path university of agriculture and technology.

[104] Andrews D.J., Kumar K.A. (1996). Use of the West African pearl millet landrace Iniadi in cultivar development. Plant Gen. Res. News..

[105] Chen L., Liu Y.-G. (2014). Male Sterility and Fertility Restoration in Crops. Annu. Rev. Plant. Biol..

[106] Chang Z., Chen Z., Wang N., Xie G., Lu J., Yan W., Zhou J., Tang X., Deng X.W. (2016). Construction of a male sterility system for hybrid rice breeding and seed production using a nuclear male sterility gene. Proc. Natl. Acad. Sci. USA.

[107] Rao M.K., Devi K.U. (1983). Variation in expression of genic male sterility in pearl millet. J. Hered..

[108] Yadav D., Gupta S.K., Kulkarni V.N., Rai K.N., Behl R.K. (2010). Inheritance of A1system of cytoplasmic-nuclear male sterility in pearl millet [Pennisetum glaucum(L). R. Br.]. Cereal. Res. Commun..

[109] Gupta S., Rai K.N., Govindaraj M., Rao A. (2012). Genetics of fertility restoration of the A4 cytoplasmic-nuclear male sterility system in pearl millet. Czech. J. Genet. Plant. Breed..

[110] Li X.Q., Jean M., Landry B.S., Brown G.G. (1998). Restorer genes for different forms of Brassicacytoplasmic Male Sterility Map to a Single Nuclear Locus that Modifies Transcripts of Several Mitochondrial Genes. Proc. Natl. Acad. Sci. USA.

[111] Islam A., Mian M.A., Rasul G., Bashar K., Johora F.-T. (2015). Development of Component Lines (CMS, Maintainer and Restorer lines) and their Maintenance Using Diversed Cytosources of Rice. Rice Res. Open Access.

[112] Delorme V., Keen C.L., Rai K.N., Leaver C.J. (1997). Cytoplasmic-Nuclear Male Sterility in Pearl Millet: Comparative RFLP and Transcript Analyses of Isonuclear Male-Sterile Lines. Theor. Appl. Genet..

[113] Pucher A., Hash C.T., Wallace J.G., Han S., Leiser W.L., Haussmann B.I.G. (2018). Mapping a male-fertility restoration locus for the A4 cytoplasmic-genic male-sterility system in pearl millet using a genotyping-by-sequencing-based linkage map. BMC Plant. Biol..

[114] Burton G.W. (1977). Fertile Sterility Maintainer Mutants in Cytoplasmic Male Sterile Pearl Millet 1. Crop. Sci..

[115] Burton G.W. (1980). Pearl Millet. Hybridization of Crop Plants.

[116] Hanna W.W., Wells H.D., Burton G.W., Monson W.G. (1988). Registration of ÔTifleaf 2Õ pearl millet. Crop Sci..

[117] Hanna W.W., Hill G.M., Gates R.N., Wilson J., Burton G.W. (1997). Registration of ‘Tifleaf 3’ Pearl Millet. Crop. Sci..

[118] Gulia S.K., Wilson J.P., Carter J., Singh B.P. (2007). Progress in grain pearl millet research and market development. Issues New Crops New Uses.

[119] Hanna W., Wilson J., Timper P. (2005). Registration of Pearl Millet Parental Lines Tift 99D2A1/B1. Crop sci..

[120] Hanna W., Wilson J., Timper P. (2005). Registration of Pearl Millet Parental Line Tift 454. Crop. Sci..

[121] Burton G.W., Athwal D.S. (1969). Registration of Pearl Millet Inbreds Tift 239DB2 and Tift 239DA2 1 (Reg. No. PL 5, PL 6). Corp Sci..

[122] Athwal D.S. (1965). Hybrid bajra-1 marks a new era. Ind. Farm..

[123] Aken’Ova M.E., Chheda H.R. (1981). A New Source of Cytoplasmic—Genic Male Sterility in Pearl Millet 1. Crop. Sci..

[124] Appadurai R., Raveendran T.S., Nagarajan C. (1982). A new male-sterility system in pearl millet. Indian J. Genet. Plant Breed..

[125] Rai K.N. (1995). A new cytoplasmic-nuclear male sterility system in pearl millet. Plant Breed..

[126] Marchais L., Pernes J. (1985). Genetic divergence between wild and cultivated pearl millets (Pennisetum typhoides): 1. Male sterility. Zeitschrift fur Pflanzenzüchtung.

[127] Hanna W.W. (1989). Characteristics and Stability of a New Cytoplasmic-Nuclear Male-Sterile Source in Pearl Millet. Crop. Sci..

[128] Govindaraj M., Rai K.N., Cherian B., Pfeiffer W.H., Kanatti A., Shivade H. (2019). Breeding Biofortified Pearl Millet Varieties and Hybrids to Enhance Millet Markets for Human Nutrition. Agriculture.

[129] Khairwal I.S., Yadav O.P. (2005). Pearl millet (Pennisetum glatlcum) improvement in India-retrospect and prospects. Indian Agric. Sci..

[130] Yadav O.P., Khairwal I.S. (2007). Progress towards developing dual-purpose cultivars of pearl millet (Pennisetum glaucum) in India. Indian J. Agric. Sci..

[131] Gill K.S., Phul P.S., Jindla L.N. (1975). New bajra hybrids resistant to the downy mildew green ear disease. Seed Forms.

[132] Pokhriyal S.C., Unnikrishnan K.V., Singh B., Dass R., Patil R.R. (1976). Combining ability of downy mildew resistant lines in pearl millet. Indian J. Genet. Plant Breed.

[133] Kumar P., Sharma V.K., Prasad B.D. (2015). Characterization of maintainer and restorer lines for wild abortive cytoplasmic male sterility in indica rice (’Oryza sativa’L.) using pollen fertility and microsatellite (SSR) markers. Aust. J. Crop. Sci..

[134] Amiribehzadi A., Satyavathi C.T. (2012). Fertility restoration studies in different cytoplasms of pearl millet [Pennisetum glaucum (L.) R. BR.]. Ann. Agric. Sci..

[135] Shivhare R., Lata C. (2017). Exploration of Genetic and Genomic Resources for Abiotic and Biotic Stress Tolerance in Pearl Millet. Front. Plant Sci..

[136] Lachagari V.B.R., Gupta R., Lekkala S.P., Mahadevan L., Kuriakose B., Chakravartty N., Katta A.V.S.K.M., Santhosh S., Reddy A.R., Thomas G. (2019). Whole Genome Sequencing and Comparative Genomic Analysis Reveal Allelic Variations Unique to a Purple Colored Rice Landrace (Oryza sativa ssp. indica cv. Purpleputtu). Front. Plant Sci..

[137] Alaux M., Rogers J., Letellier T., Flores R., Alfama F., Pommier C., Mohellibi N., Durand S., Kimmel E., International Wheat Genome Sequencing Consortium (2018). Linking the International Wheat Genome Sequencing Consortium bread wheat reference genome sequence to wheat genetic and phenomic data. Genome Biol..

[138] Varshney R.K., Song C., Saxena R.K., Azam S., Yu S., Sharpe A.G., Cannon S., Baek J., Rosen B.D., Tar’An B. (2013). Draft genome sequence of chickpea (Cicer arietinum) provides a resource for trait improvement. Nat. Biotechnol..

[139] Kreplak J., Madoui M.-A., Cápal P., Novak P., Labadie K., Aubert G., Bayer P.E., Gali K.K., Syme R., Main R. (2019). A reference genome for pea provides insight into legume genome evolution. Nat. Genet..

[140] Varshney R.K., Chen W., Li Y., Bharti A.K., Saxena R.K., Schlueter J., A Donoghue M.T., Azam S., Fan G., Whaley A.M. (2011). Draft genome sequence of pigeonpea (Cajanus cajan), an orphan legume crop of resource-poor farmers. Nat. Biotechnol..

[141] Cooper E.A., Brenton Z.W., Flinn B.S., Grimwood J., Shu S., Flowers D., Luo F., Wang Y., Xia P., Barry K. (2019). A new reference genome for Sorghum bicolor reveals high levels of sequence similarity between sweet and grain genotypes: Implications for the genetics of sugar metabolism. BMC Genom..

[142] Meyer S., Pospisil H., Scholten S. (2006). Heterosis associated gene expression in maize embryos 6 days after fertilization exhibits additive, dominant and overdominant pattern. Plant. Mol. Biol..

[143] Zhang X., Wang Y., Yan Y., Peng H., Long Y., Zhang Y., Jiang Z., Liu P., Zou C., Peng H. (2019). Transcriptome sequencing analysis of maize embryonic callus during early redifferentiation. BMC Genom..

[144] Wang C., Tariq R., Ji Z., Wei Z., Zheng K., Mishra R., Zhao K. (2019). Transcriptome analysis of a rice cultivar reveals the differentially expressed genes in response to wild and mutant strains of Xanthomonas oryzae pv. oryzae. Sci. Rep..

[145] Yan L., Liu Z., Xu H., Zhang X., Zhao A., Liang F., Xin M., Peng H., Yao Y., Sun Q. (2018). Transcriptome analysis reveals potential mechanisms for different grain size between natural and resynthesized allohexaploid wheats with near-identical AABB genomes. BMC Plant. Biol..

[146] Jaikishan I., Rajendrakumar P., Hariprasanna K., Bhat B.V. (2018). Gene Expression Analysis in Sorghum Hybrids and Their Parental Lines at Critical Developmental Stages in Relation to Grain Yield Heterosis by Exploiting Heterosis-Related Genes from Major Cereals. Plant Mol. Biol. Rep..

[147] Zhang H., Hall N., Goertzen L.R., Chen C.Y., Peatman E., Patel J., McElroy J.S. (2019). Transcriptome Analysis Reveals Unique Relationships Among Eleusine Species and Heritage of Eleusine coracana. G3 Genes|Genomes|Genet..

[148] Hiremath P.J., Farmer A., Cannon S.B., Woodward J., Kudapa H., Tuteja R., Kumar A., Bhanuprakash A., Mulaosmanovic B., Gujaria N. (2011). Large-scale transcriptome analysis in chickpea (Cicer arietinum L.), an orphan legume crop of the semi-arid tropics of Asia and Africa. Plant Biotechnol. J..

[149] Dudhate A., Shinde H., Tsugama D., Liu S., Takano T. (2018). Transcriptomic analysis reveals the differentially expressed genes and pathways involved in drought tolerance in pearl millet [Pennisetum glaucum (L.) R. Br]. PLoS ONE.

[150] Singh R.B., Singh B., Singh R.K. (2019). Development of potential dbEST-derived microsatellite markers for genetic evaluation of sugarcane and related cereal grasses. Ind. Crop. Prod..

[151] Diack O., Kane N.A., Berthouly-Salazar C., Gueye M.C., Diop B.M., Fofana A., Sy O., Tall H., Zekraoui L., Piquet M. (2017). New Genetic Insights into Pearl Millet Diversity As Revealed by Characterization of Early- and Late-Flowering Landraces from Senegal. Front. Plant. Sci..

[152] Anuradha N., Satyavathi C.T., Bharadwaj C., Nepolean T., Sankar S.M., Singh S.P., Meena M.C., Singhal T., Srivastava R.K. (2017). Deciphering Genomic Regions for High Grain Iron and Zinc Content Using Association Mapping in Pearl Millet. Front. Plant Sci..

[153] Taunk J., Rani A., Yadav N.R., Yadav D.V., Yadav R.C., Raj K., Kumar R., Yadav H.P. (2018). Molecular breeding of ameliorating commercial pearl millet hybrid for downy mildew resistance. J. Genet..

[154] Serba D.D., Muleta K.T., Amand P.S., Bernardo A., Bai G., Perumal R., Bashir E. (2019). Genetic Diversity, Population Structure, and Linkage Disequilibrium of Pearl Millet. Plant Genome.

[155] Srivastava R.K., Singh R.B., Pujarula V.L., Bollam S., Pusuluri M., Chellapilla T.S., Yadav R.S., Gupta R.K. (2020). Genome-Wide Association Studies and Genomic Selection in Pearl Millet: Advances and Prospects. Front. Genet..

[156] Liang Z., Gupta S.K., Yeh C.-T., Zhang Y., Ngu D.W., Kumar R., Patil H.T., Mungra K., Yadav D.V., Rathore A. (2018). Phenotypic Data from Inbred Parents Can Improve Genomic Prediction in Pearl Millet Hybrids. G3 Genes|Genomes|Genet..

[157] Liu C.J., Witcombe J.R., Pittaway T.S., Nash M., Hash C.T., Busso C.S., Gale M.D. (1994). An RFLP-based genetic map of pearl millet (Pennisetum glaucum). Theor. Appl. Genet..

[158] Devos K.M., Pittaway T.S., Busso C.S., Gale M.D., Witcombe J.R., Hash C.T. (1995). Molecular tools for the pearl millet nuclear genome. Int. Sorghum Millets Newslett..

[159] Rajaram V., Thirunavukkarasu N., Senthilvel S., Varshney R.K., Vadez V., Srivastava R.K., Shah T., Supriya A., Kumar S., Kumari B.R. (2013). Pearl millet [Pennisetum glaucum (L.) R. Br.] consensus linkage map constructed using four RIL mapping populations and newly developed EST-SSRs. BMC Genom..

[160] Allouis S., Qi X., Lindup S., Gale M.D., Devos K.M. (2001). Construction of a BAC library of pearl millet, Pennisetum glaucum. Theor. Appl. Genet..

[161] Kumar S., Hash C.T., Singh G., Basava R.K., Srivastava R.K. (2020). Identification of polymorphic SSR markers in elite genotypes of pearl millet and diversity analysis. Ecol. Genet. Genom..

[162] Supriya A., Senthilvel S., Nepolean T., Eshwar K., Rajaram V., Shaw R., Hash C.T., Kilian A., Yadav R.C., Narasu M.L. (2011). Development of a molecular linkage map of pearl millet integrating DArT and SSR markers. Theor. Appl. Genet..

[163] Sehgal D., Rajaram V., Armstead I., Vadez V., Yadav Y.P., Hash C.T., Yadav R. (2012). Integration of gene-based markers in a pearl millet genetic map for identification of candidate genes underlying drought tolerance quantitative trait loci. BMC Plant. Biol..

[164] Singh R.B., Singh B., Singh R.K. (2019). Cross-taxon transferability of sugarcane expressed sequence tags derived microsatellite (EST-SSR) markers across the related cereal grasses. J. Plant Biochem. Biotechnol..

[165] Gurung D.B., George M.L.C., DeLaCruz Q.D. (2009). Determination of Heterotic Groups in Nepalese Yellow Maize Populations. Nepal J. Sci. Technol..

[166] Singh S., Gupta S.K. (2019). Formation of heterotic pools and understanding relationship between molecular divergence and heterosis in pearl millet [Pennisetum glaucum (L.) R. Br.]. PLoS ONE.

[167] Teklewold A., Becker H.C. (2005). Comparison of phenotypic and molecular distances to predict heterosis and F1 performance in Ethiopian mustard (Brassica carinata A. Braun). Theor. Appl. Genet..

[168] (2010). 168. Basavaraj, G; Rao, P.P; Bhagavatula, S; Ahmed, W. Availability and utilization of pearl millet in India. SAT eJournal.

[169] Singh A.M., Rana M.K., Singh S., Kumar S., Kumar D., Arya L. (2013). Assessment of genetic diversity among pearl millet [Pennisetum glaucum (L) R Br] cultivars using SSR markers. Range Manag. Agrofor..

[170] Singh S., Gupta S.K., Thudi M., Das R.R., Vemula A., Garg V., Varshney R.K., Rathore A., Pahuja S.K., Yadav D.V. (2018). Genetic Diversity Patterns and Heterosis Prediction Based on SSRs and SNPs in Hybrid Parents of Pearl Millet. Crop. Sci..

[171] Bhardwaj R., Garg T., Malik E.A., Vikal Y., Sohu R.S., Gupta S.K. (2018). Genetic divergence studies in pearl millet (Pennisetum glaucum L. (R.) Br.) inbred lines. Indian J. Genet. Plant. Breed..

[172] Kapadia V.N. (2016). Estimation of Heterosis for Yield and Its Relevant Traits in Forage Pearl Millet [*Pennisetum glaucum* LR Br.]. Int. J. Agric. Sci..

[173] Satyavathi C.T., Tiwari S., Bharadwaj C., Rao A.R., Bhat J., Singh S.P. (2013). Genetic diversity analysis in a novel set of restorer lines of pearl millet [*Pennisetum glaucum* (L.) R. Br] using SSR markers. Vegetos.

[174] Sumanth M., Sumathi P., Vinodhana N.K., Sathya M. (2013). Assessment of genetic distance among the inbred lines of pearl millet (Pennisetum glaucum (L.) R. Br.) using SSR markers. Int. J. Biotechnol. Allied Fields.

[175] Stich B., Haussmann B.I., Pasam R.K., Bhosale S., Hash C.T., Melchinger A.E., Parzies H.K. (2010). Patterns of molecular and phenotypic diversity in pearl millet [Pennisetum glaucum (L.) R. Br.] from West and Central Africa and their relation to geographical and environmental parameters. BMC Plant. Biol..

[176] Spindel J., Begum H., Akdemir D., Virk P., Collard B., Redona E., Atlin G., Jannink J.L., McCouch S.R. (2015). Genomic selection and association mapping in rice (Oryza sativa): Effect of trait genetic architecture, training population composition, marker number and statistical model on accuracy of rice genomic selection in elite, tropical rice breeding lines. PLoS Genet..

[177] Zhong S., Dekkers J.C.M., Fernando R.L., Jannink J.-L. (2009). Factors Affecting Accuracy From Genomic Selection in Populations Derived From Multiple Inbred Lines: A Barley Case Study. Genetics.

[178] Heffner E.L., Sorrells M.E., Jannink J. (2009). Genomic Selection for Crop Improvement. Crop. Sci..

[179] Crossa J., Campos G.D.L., Pérez-Rodríguez P., Gianola D., Burgueño J., Araus J.L., Makumbi D., Singh R.P., Dreisigacker S., Yan J. (2010). Prediction of Genetic Values of Quantitative Traits in Plant Breeding Using Pedigree and Molecular Markers. Genetics.

[180] Poland J., Endelman J.B., Dawson J., Rutkoski J., Wu S., Manès Y., Dreisigacker S., Crossa J., Sanchez-Villeda H., Sorrells M. (2012). Genomic Selection in Wheat Breeding using Genotyping-by-Sequencing. Plant. Genome.

[181] Ornella L., Singh S., Pérez-Rodríguez P., Burgueño J., Singh R., Tapia E., Bhavani S., Dreisigacker S., Braun H.-J., Mathews K. (2012). Genomic Prediction of Genetic Values for Resistance to Wheat Rusts. Plant. Genome.

[182] Muleta K.T., Pressoir G., Morris G.P. (2018). Optimizing Genomic Selection for a Sorghum Breeding Program in Haiti: A Simulation Study. G3 Genes|Genomes|Genet..

[183] Meuwissen T.H., Hayes B.J., E Goddard M. (2001). Prediction of total genetic value using genome-wide dense marker maps. Genetics.

[184] Lorenz A.J., Smith K., Jannink J.-L. (2012). Potential and Optimization of Genomic Selection for Fusarium Head Blight Resistance in Six-Row Barley. Crop. Sci..

[185] Heffner E.L., Lorenz A.J., Jannink J., Sorrells M.E. (2010). Plant Breeding with Genomic Selection: Gain per Unit Time and Cost. Crop. Sci..

[186] Crossa J., Pérez-Rodríguez P., Cuevas J., Montesinos-Lopez O.A., Jarquin D., Campos G.D.L., Burgueño J., González-Camacho J.M., Elizalde S.P., Beyene Y. (2017). Genomic Selection in Plant Breeding: Methods, Models, and Perspectives. Trends Plant Sci..

